# Efficacy of 0.5% timolol maleate gel in the management of infantile hemangiomas

**DOI:** 10.12669/pjms.40.2(ICON).8983

**Published:** 2024-01

**Authors:** Aqsa Mazhar, Afza Naureen, Yousuf Abd Mallick, Lubna Samad

**Affiliations:** 1 Aqsa Mazhar, MBBS, FCPS Vascular Anomalies Center, The Indus Hospital And Health Network, Karachi, Pakistan; 2 Afza Naureen Ghouse, MBBS, FCPS Department of Dermatology, The Indus Hospital And Health Network, Karachi, Pakistan; 3 Yousuf Abd Mallick, MBBS, FCPS Department of Dermatology, Liaquat College of Medicine and Dermatology, Karachi, Pakistan; 4 Dr. Lubna Samad, MBBS, MRCS, FCPS Director Global Surgery, Interactive Research and Development (IRD) Global, Karachi, Pakistan

**Keywords:** Infantile hemangiomas, Timolol gel, Propranolol

## Abstract

**Objective:**

This study aimed to evaluate the efficacy and safety of 0.5% timolol maleate gel, reported as an efficacious option for management of infantile hemangiomas (IH) in children.

**Methods:**

A retrospective study was conducted among patients diagnosed with IH from January 2019 to December 2021. All patients were treated 0.5% timolol gel. Data parameters, including photographs, at baseline and the final or most recent follow-up visit, were reviewed. Outcomes based on photographic assessment were categorized as excellent, good, fair or poor.

**Results:**

Sixty-four children with 76 lesions were enrolled. Median age was eight months (two months to 36 months) with most lesions (75.0%) presenting during the first year of life. Female preponderance (84.4%) was seen and the cervicofacial region was most commonly involved (52.6%). The majority of lesions (54, 84.4%) were solitary and most were treatment naïve (n=61, 80.3%). Excellent, good, fair, and poor responses were seen in 24 (31.5%), 39 (51.3%), 6 (7.9%), and 7 (9.2%) lesions. No complications were seen and no statistically significant difference was observed with respect to gender, age group, region involved and treatment naïve versus previously treated patients.

**Conclusion:**

Timolol maleate 0.5% gel is an effective and safe treatment option for IH irrespective of location of lesion, age and history of prior treatment.

## INTRODUCTION

Infantile hemangiomas (IH) are the most commonly reported benign tumors of infancy. Western literature reported a high incidence of 5-10% of all infants affected^1^ as compared to a much lower incidence of 0.1–0.28% in the Indian subcontinent.^2^ IH are more common in females and low birth weight infants, and their incidence varies among studies due to ethnical, geographical, racial, and environmental differences.^3^,^4^ Nearly 50% of neonates have a premonitory mark evident at birth which can manifest as a telangiectatic macule, a pale macule, an erythematous macule, or less commonly as a bruise, scratch, or blurred swelling.

It is characterized by three phases of growth namely: proliferative, involuting, and involuted.^2^ The proliferative phase is responsible for symptomatic entanglement. Owing to their natural history, IH has a spontaneous involution rate of approximately 10% per year.^4^ Nonetheless, about 50 to 60% of IH need treatment to halt their proliferation phase and preserve the normal function of affected anatomical structures and prevent serious disfigurement or complications.^5^,^6^ IH have been treated with oral medication (steroids, beta-blockers), injection sclerotherapy, laser treatment, and surgical excision with various degrees of success.^2^ The choice to start a particular mode of treatment depends upon the age of the patient, the site and size of the lesion, parental preferences, and tolerance to a particular mode of treatment. Using a multidisciplinary approach, optimal results can be achieved using individualized treatment regimens that are well-suited to the needs of patients and parents.

Non-selective beta-adrenergic receptor blockers (β-blockers) emerged as a good therapeutic option for IH in 2008, when Leaute-Labreze et al. reported incidental regression of a facial IH in a child who was treated with propranolol for obstructive hypertrophic cardiomyopathy.^7^ Over the past decade, oral propranolol has been used extensively with good results.^8^ However, the development of side effects including bradycardia, hypoglycemia, dry skin and mouth, nausea, diarrhea, sleep disturbances, and bronchospasm leading to breathing difficulties in some cases has limited its use. A side effect rate of 17% has been reported in a case series of 100 patients by Danielle H et al.^9^

In 2010, Pope and Chakkittakandiyil described 0.5% topical timolol gel as a safe and effective treatment in a series of six patients with head and neck IH.^10^ Since then, the use of topical β-blockers in the treatment of IH has been widely reported in the literature, with comparable therapeutic efficacy and little to no systemic adverse effects.^8^,^11^ The authors of this study add to the reported literature their experience of treating IH with 0.5% topical timolol maleate gel in 64 children.

Though widely reported in the literature globally, the efficacy and safety of timolol in the gel form have not been defined in our region. We hope this study will encourage local formulation and use of timolol gel so that better efficacy can be achieved. Our study aimed to assess the clinical effectiveness and safety of topical 0.5% timolol maleate gel in treating infantile hemangiomas in an outpatient setting in Karachi, Pakistan.

## METHODS

This is a single-center, retrospective study conducted at the Vascular Anomalies Center (VAC) of The Indus Hospital & Health Network (IHHN), Karachi, Pakistan, from January 2019 to December 2021.

### Inclusion criteria

All children (36 months and below) with a primary diagnosis of superficial, uncomplicated infantile hemangioma who had either completed treatment or were under treatment with a minimum follow-up period of six months were included in the study. Patients on oral propranolol treatment who developed side effects and were started on topical timolol treatment were also included in this study.

### Exclusion criteria

Children with congenital hemangioma, ulcerated and/or infected hemangiomas, associated syndromic malformations, and multisystem involvement were excluded.

### Ethical Approval

Ethical approval from the Institutional Review Board was obtained (IRD_IRB_2020_02_004).

Topical 0.5% timolol maleate gel was prepared by IHHN pharmacy and prescribed to all enrolled patients. Twice daily topical application of one to two drops of timolol gel was advised, so that the entire surface of the lesion was covered, and then left to dry. This was preferably done at a time when the child was asleep. Three-monthly visits were advised, and a helpline mobile number was given to them so that they can contact the VAC team if required.

### Questionnaire

A predefined questionnaire was used to collect demographic and clinical data for all patients managed at the VAC clinic, including age at presentation, gender, birth history, time of appearance of lesion, number, and location of the lesion (cervicofacial, truncal, or extremity), family history, and prior interventions. Progress using photographic documentation was also routinely recorded with parental consent. Data parameters, including photographs, at baseline and the final or most recent follow-up visit, were reviewed for all included patients. Study numbers were assigned. Photographs taken at the corresponding clinic visit were reviewed, and the percentage decrease in the size of the lesion as well as fading of color were noted. Response to the treatment was categorized into excellent, good, fair, and poor [Table-I].

**Table-I:** Response categories following treatment.

Categories	Reduction in size	Fading of color
Excellent response*	>90% size reduction	Complete fading of the color
Good response	>50% to 90% size reduction	Significant fading of color
Fair response	30 to 50% reduction	Minimal fading of color
Poor response	< 30% reduction	No response

* If complete fading occurred with <90% size reduction then it will be taken as “Excellent response”.

### Statistical analysis

Stratification was done concerning the age of lesions (up to six months, 7 to 12 months, and >12 months) and the treatment status of lesions. Lesions were classified into two groups; ‘treatment naïve’ (Group A) and ‘prior-treated lesions’ (Group B).

Data were entered and analysed by using version 26.0 of a statistical package of social sciences (SPSS) software. Median (IQR) was computed for quantitative variables while frequency and percentages were computed for categorical variables. Pearson Chi-Square or Fisher’s exact tests were applied as appropriate to assess the association of gender, age group, type of lesion, prior treatment status, and category of patients with response to treatment. P-value ≤0.05 was considered statistically significant.

## RESULTS

Sixty-four patients with infantile hemangiomas were enrolled in this study with a majority n=54 (84.4%) presenting with solitary lesions while ten patients (15.6%) had two or three lesions; thus, a total of 76 lesions were included in the analysis. The median age of children was eight months. A marked female predominance (n=54, 84.4%) was seen with a female-to-male ratio of 5:1. Most patients presented during the first year of life (n=48, 75%), while 16(25%) were between one to three years at initial presentation to VAC clinic. The affected children were born full-term or preterm in 60 (93.75%) and four (6.25%) cases respectively. A family history of IH was elicited in three (4.6%) cases: two siblings and one first cousin was affected.

A precursor sign was seen at birth in forty-nine (64.4%) children, which manifested as a red dot (n=32, 65.3%), a red patch (n=14, 28.5%), or bluish discoloration (n=3, 6.1%). A total of 74 (97.3%) lesions were established as IH in the first four weeks of post-natal life. Commonly involved areas were cervicofacial (n=40, 52.6%), trunk (n=21, 27.6%), and extremities (n=15, 19.7%) in IH lesions. The majority of lesions were treatment naïve (n=61, 80.3%).

In our study, 15(23.4%) patients who received other treatment before initiation of timolol gel including propranolol were 11(73.3%), pulse dyed laser (PDL) three (20.0%), and injection sclerotherapy (IST) with Bleomycin was one patient (6.6%). In the propranolol-treated cases, an initial response was noted in all cases, but treatment was changed due to side effects (diarrhea and hypoglycemia) in three children and due to parental preference in eight children. The three children who received PDL treatment showed an early response but were switched to timolol due to parental preference. The child who underwent IST at another facility did not show any treatment response, and was converted to timolol gel at VAC.

Excellent, good, fair, and poor responses were seen in 24 (31.5%), 39 (51.3%), 6 (7.9%), and seven (9.2%) lesions on clinical and photographic assessment respectively [Table-II]. A statistically significant difference was found for gender (p-value 0.043) while no association in outcome concerning age groups, location, and category of lesions was noted [Table-II].

**Table-II:** Association of assessment outcomes of lesions with independent variables.

	Excellent n (%)	Good n (%)	Fair n (%)	Poor n (%)	Total n (%)	P value
***Gender***						
Male	6(25)	4(10.3)	3(50)	-	13(17.1)	0.043^*ꝉ^
Female	18(75)	35(89.7)	3(50)	7(100)	63(82.9)	
Total	24(100)	39(100)	6(100)	7(100)	76(100)	
***Age group (months)***						
≤6	8(33.3)	19(48.7)	4(66.7)	5(71.4)	36(47.4)	0.154^ꝉ^
7-12	11(45.8)	9(23.1)	2(33.3)	-	22(28.9)	
>12	5(20.8)	11(28.2)	-	2(28.6)	18(23.7)	
Total	24(100)	39(100)	6(100)	7(100)	76(100)
***Location***						
Cervicofacial	14(58.3)	20(51.3)	3(50)	3(42.9)	40(52.6)	0.386^ꝉ^
Trunk	5(20.8)	6(15.4)	3(50)	1(14.3)	15(19.7)	
Extremity	5(20.8)	13(33.3)	-	3(42.9)	21(27.6)	
Total	24(100)	39(100)	6(100)	7(100)	76(100)
***Treatment Groups***						
Treatment Naïve	19(79.2)	29(74.4)	6(100)	7(100)	61(80.3)	0.383^ꝉ^
Prior Treated	5(20.8)	10(25.6)	-	-	15(19.7)	
Total	24(100)	39(100)	6(100)	7(100)	76(100)	

* P <0.05, ꝉ Fisher exact test.

**Fig.1:**
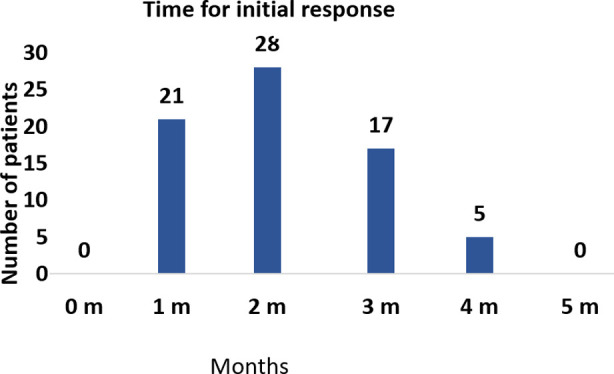
Response of treatment to Timolol maleate 0.5% gel.

The mean ± SD duration of treatment was 9.38±3.42 months with a mean±SD duration of follow-up was 10.38±3.92 months. Response was visible as early as in one month in 27.6%, with all but five lesions showing a response within four months of initiating treatment (Fig.1). The five lesions with poor response were switched to oral propranolol and all five demonstrated a visible response within six months. Timolol gel was equally effective in Group A and B, with no statistically significant difference seen (p-value 0.383). In the treatment completed group (n=44, 68.75%), no recurrence was noted at the six month follow-up. No systemic or local adverse effects were recorded for any enrolled child.

## DISCUSSION

Our study evaluating the efficacy and treatment outcomes of topical timolol gel in a group of 64 children is the first report from Pakistan to document use of timolol gel in children with IH. A marked improvement after topical timolol treatment is demonstrated, with 31.5% and 51.3% children showing an excellent or good response respectively.

Topical timolol was initially proposed as a treatment option for IH^10^ due to concerns over the systemic side effects of oral beta-blockers. The mode of action of beta-blockers remains unclear, but vasoconstriction, down-regulation of angiogenic factors such as VEGF and bFGF and up-regulation of apoptosis of capillary endothelial cells may be responsible for the inhibition of growth, thus promoting regression of superficial IH.^12^,^13^ In this study, we found a significant response of treatment in 93.4% of lesions within four months of initiating topical timolol gel treatment. This compares well with literature, where a 91.4% response was seen in the same time frame.^14^ As compared to this, treatment response ranging from 59% to 83.8% has been reported with oral propranolol treatment, with a longer response time of between 24 to 52 weeks noted^12^,^15^ as compared to our patient series. The lower risk of systematic side effects with topical application of beta blocker is an added advantage, whereby timolol gel can simply be applied by parents without need for work-up or close monitoring.

The majority of our study participants were female (5:1) which is similar to other reports^1^,^16^-^19^ We found better response to timolol treatment in females; other authors have noted similar efficacy of timolol gel in both genders.^16^,^17^,^20^ Our findings may be biased by the marked female preponderance; since the sample size is relatively small, stratification based on gender is likely to provide erroneous results.

In our study, the median age of children was eight months (range 2-36 months) at the time of initial presentation, with 52.6% older than six months. In other studies, topical timolol is usually initiated within the first six months of life,^1^,^16^,^17^,^19^,^21^ but we have demonstrated comparable success in treating older children. This is of particular importance in low-income settings like ours where affected children often present late to specialist facilities. Similarly, even those children who had received other treatment modalities were responsive to topical timolol. Moreover, no side effects were noted in our cohort, which is a major advantage since patients often come to our clinic from remote areas making frequent follow-up visits difficult.

The study design is retrospective which allowed us to follow patients on treatment. Since all patients were enrolled using a standardized database and followed after initiation of treatment, we were able to systematically document response patterns.

### Limitations

The absence of controls is a limitation to our approach; while a no-treatment control group would be difficult to justify ethically, we could have included a second arm of children on oral propranolol, thus allowing direct comparison of outcomes between the two most used medical treatment modalities for IH. Although the VAC Clinic is the largest of its kind in the country, our patient numbers could have been increased by recruiting at multiple centers across the country. We were unable to do so due to financial constraints, and also due to the absence of structured vascular anomalies services and systematic data collection at other large-volume children’s hospitals. We do not have resources to study the histopathological and molecular level patterns of IH and this limits our ability to classify different stages and subsets of IH against treatment responses.

## CONCLUSION

Timolol maleate 0.5% gel is an effective, safe, and well-tolerated treatment modality for IH. Response appears to be more rapid in females. Topical timolol appears to be effective irrespective of location of lesion, age and history of prior treatment, and is therefore recommended as the first line treatment for uncomplicated infantile hemangioma.

### Authors’ Contribution:

**AM, AF, YM** conceived and designed the concept, collected data, drafted and edited the manuscript and is responsible for the integrity of the research.

**LS** reviewed and approved the final version of the manuscript.
